# Disentangling the Common Variance of Perfectionistic Strivings and Perfectionistic Concerns: A Bifactor Model of Perfectionism

**DOI:** 10.3389/fpsyg.2017.00160

**Published:** 2017-02-13

**Authors:** Jana C. Gäde, Karin Schermelleh-Engel, Andreas G. Klein

**Affiliations:** Department of Psychology, Goethe UniversityFrankfurt, Germany

**Keywords:** Perfectionism, Perfectionism Inventory, perfectionistic strivings, perfectionistic concerns, bifactor model, confirmatory factor analysis, construct validation

## Abstract

Perfectionism nowadays is frequently understood as a multidimensional personality trait with two higher-order dimensions of perfectionistic strivings and perfectionistic concerns. While perfectionistic concerns are robustly found to correlate with negative outcomes and psychological malfunctioning, findings concerning the outcomes of perfectionistic strivings are inconsistent. There is evidence that perfectionistic strivings relate to psychological maladjustment on the one hand but to positive outcomes on the other hand as well. Moreover, perfectionistic strivings and perfectionistic concerns frequently showed substantial overlap. These inconsistencies of differential relations and the substantial overlap of perfectionistic strivings and perfectionistic concerns raise questions concerning the factorial structure of perfectionism and the meaning of its dimensions. In this study, several bifactor models were applied to disentangle the common variance of perfectionistic strivings and perfectionistic concerns at the item level using Hill et al.’s (2004) Perfectionism Inventory (PI). The PI measures a broad range of perfectionism dimensions by four perfectionistic strivings and four perfectionistic concerns subscales. The bifactor-(*S* – 1) model with one general factor defined by concern over mistakes as the reference facet, four specific perfectionistic strivings factors, and three specific perfectionistic concerns factors showed acceptable fit. The results revealed a clear separation between perfectionistic strivings and perfectionistic concerns, as the general factor represented concern over mistakes, while the perfectionistic strivings factors each explained a substantial amount of reliable variance independent of the general factor. As a result, factor scores of the specific perfectionistic strivings factors and the general factor had differential relationships with achievement motivation, neuroticism, conscientiousness, and self-efficacy that met with theoretical expectations, while results for manifest subscale scores were ambiguous. Our results question the existence of reliable sub-constructs of perfectionistic concerns independent of the general factor when defined by concern over mistakes.

## Introduction

A lot of research has been conducted on the nature and structure of perfectionism over the past 35 years. Early conceptions defined perfectionism as a unidimensional and merely dysfunctional personality trait (Burns, 1980) or differentiated between a normal and a neurotic form of perfectionism (Hamachek, 1978). For about 25 years, a multidimensional definition of perfectionism has been popular. The dimensions of this multidimensional construct vary considerably depending on the underlying perfectionism concepts (Frost et al., 1990; Hewitt and Flett, 1991; Slaney et al., 2001; Hill et al., 2004). However, theoretical and empirical evidence has suggested that these manifold perfectionism dimensions can be assigned to two higher-order factors of perfectionistic strivings and perfectionistic concerns (Frost et al., 1993; Slade and Owens, 1998; Cox et al., 2002; Bieling et al., 2004).

Perfectionistic strivings comprise striving for flawlessness and setting unrealistic high performance standards (personal standards, Frost et al., 1990; self-oriented perfectionism, Hewitt and Flett, 1991; standards, Slaney et al., 2001, striving for excellence, Hill et al., 2004), order and organization (Frost et al., 1990; Slaney et al., 2001; Hill et al., 2004), planning ahead (planfulness, Hill et al., 2004), and setting unrealistic high standards of performance for significant others (other-oriented perfectionism, Hewitt and Flett, 1991; high standards for others, Hill et al., 2004).

Perfectionistic concerns comprise an overly critical evaluation of oneself (concern over mistakes, Frost et al., 1990; Hill et al., 2004), a perceived difference between one’s personal standards and one’s actual performance (discrepancy, Slaney et al., 2001), doubts about actions (Frost et al., 1990), adverse reactions to failure such as rumination over mistakes (Hill et al., 2004), seeking validation from others and being sensitive to criticism (need for approval, Hill et al., 2004), and perceived parental expectations and pressure (Frost et al., 1990; Hill et al., 2004).

Numerous studies investigated the factorial structure of perfectionism and found support for two higher-order dimensions (e.g., Cox et al., 2002; Hill et al., 2004). The two-factorial structure was found for single measures (Haase and Prapavessis, 2004; Cruce et al., 2012; Stoeber and Damian, 2014) as well as for simultaneous analysis of different measures (Dunkley et al., 2012). Slade and Owens (1998) developed a dual process model of perfectionism based on reinforcement theory to explain these higher-order factors in terms of achievement motivation. According to their model, perfectionistic strivings are positively correlated with hope for success, whereas perfectionistic concerns are positively correlated with fear of failure.

There is consistent evidence that perfectionistic concerns are associated with a variety of psychiatric symptoms and psychological maladjustment (Frost et al., 1993; Hewitt et al., 1996; DiBartolo et al., 2008), while perfectionistic strivings have also been associated with desired outcomes, such as higher levels of self-efficacy and performance (Chufar, 2013). The association between perfectionistic strivings and positive outcomes became more apparent when controlling for perfectionistic concerns (e.g., Dunkley et al., 2012; see Stoeber and Otto, 2006, for an overview and review of the outcomes of perfectionistic strivings). There is an ongoing debate whether perfectionistic strivings per se are unproblematic (Dunkley et al., 2006), can buffer negative effects of perfectionistic concerns (Gaudreau and Thompson, 2010; Gaudreau, 2015) and are correlated with positive outcomes (Stoeber and Otto, 2006), or whether they are a risk factor for psychological malfunctioning besides perfectionistic concerns and contribute to negative outcomes such as eating disorders (see Egan et al., 2011, for a review of studies that found elevated levels of perfectionistic strivings in clinical samples). Frequently, substantial correlations between perfectionistic strivings and perfectionistic concerns are reported (e.g., *r* = 0.58 to 0.72, Dunkley et al., 2012; *r* = 0.69, Hill et al., 2004), which highlights a substantial construct overlap.

These findings concerning multidimensional perfectionism measures repeatedly indicate that a substantial amount of shared variance exists between perfectionistic strivings and perfectionistic concerns. These findings challenge the clear-cut distinction between perfectionistic strivings and perfectionistic concerns, which is crucial with regard to the nature and structure of perfectionism. As long as the underlying measurement model of perfectionism is not clarified such inconsistent findings are likely to occur. As a consequence either the claim for differential outcomes of perfectionistic strivings and concerns should be modified or measurement models should be taken into account that shed light onto the different sources of observed indicator variance.

**FIGURE 1:**
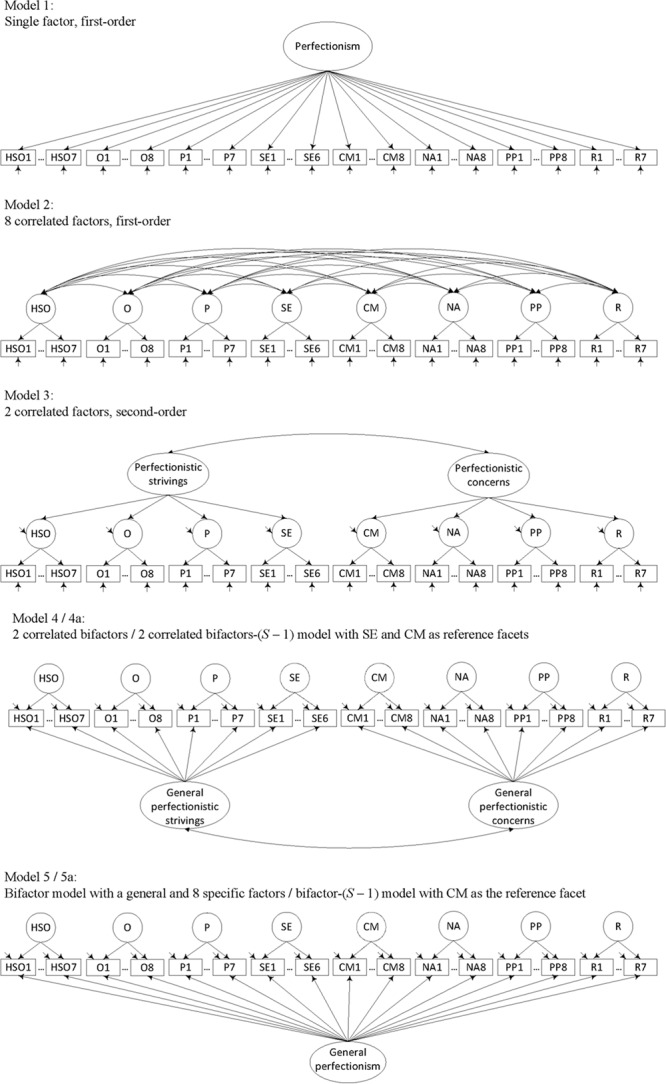
**Schematic representation of the alternative models considered in this study.** For all models, the 59 items of the PI served as indicator variables. Error terms are omitted for clarity. HSO, high standards for others; O, organization; P, planfulness; SE, striving for excellence; CM, concern over mistakes; NA, need for approval; PP, perceived parental pressure; R, rumination.

Therefore, there is a need for perfectionism models that capture the substantial amount of shared variance while preserving the multidimensional nature of perfectionism. Since correlated factor models actually are not measurement models (Reise et al., 2010), a bifactor model (see **Figure 1**) might be a useful alternative to higher-order models for explaining correlations at the item and subscale level (cf., Yung et al., 1999; Chen et al., 2006; Gignac, 2008; Reise, 2012). Bifactor models are representations of theoretical constructs that comprise one general factor and one or more specific factors. The general factor is common to all manifest variables of a scale, while the specific factors are common to subsets of manifest variables. Compared to higher-order models, bifactor models assume a general factor at the same level as the specific factors, instead of one or more superordinate factors. In bifactor models, systematic item variance is explained by a general factor and a specific factor. This specific factor explains part of the variance not accounted for by the general factor.

Several advantages are associated with bifactor models. Bifactor models can be used to evaluate whether a general factor underlies the data, to study the effects of specific factors that are independent of the general factor, and to test which specific factors predict external variables over and above the general factor. In bifactor models, the relationships between all factors, that is, general factor and specific factors, as well as the indicator variables, are apparent in the respective factor loadings (Chen et al., 2006). For hierarchical models, the Schmid and Leiman (1957) transformation procedure might be applied to estimate these relationships. However, the Schmid and Leiman (1957) transformation is based on more stringent assumptions, such as proportionality constraints, which are associated with higher-order models compared to bifactor models.

Due to the substantial construct overlap of perfectionistic strivings and perfectionistic concerns, a bifactor approach may be considered reasonable. Thus, the common variance of perfectionistic strivings and perfectionistic concerns could be captured by a general perfectionism factor, while additional specific factors should account for the unique variance not explained by the general factor. These specific factors would in turn be controlled for the impact of the general factor and would remain as pure specific perfectionism factors. Reliability of composite scores according to the general as well as the specific factors can be assessed easily. Such a measurement model is needed to disentangle the common variance of perfectionistic strivings and perfectionistic concerns. Disentangling the common variance can contribute to gaining a clearer understanding of the structure of perfectionism and to investigating the differential relations of perfectionistic strivings and perfectionistic concerns with external criteria.

### Research Questions

The objective of this study was to investigate whether a bifactor model of perfectionism might be a reasonable and viable alternative to existing factor models to capture the common variance of perfectionistic strivings and perfectionistic concerns and to assess the reliability of scale scores for this bifactor model.

Several competing factor models were evaluated using the Perfectionism Inventory (PI; Hill et al., 2004). The PI measures eight dimensions, of which six are conceptually similar to subscales of the widely used Multidimensional Perfectionism Scale MPS-F (Frost et al., 1990) and the Multidimensional Perfectionism Scale MPS-HF (Hewitt and Flett, 1991). Additionally, Hill et al. (2004) included two subscales labeled planfulness and rumination. Planfulness describes the tendency to think ahead carefully and is regarded as a positive characteristic. Rumination describes the tendency to brood over past errors, possible future mistakes, and less than perfect performance, and is regarded as a negative characteristic. Rumination was included since previous studies have found perfectionistic rumination to contribute to psychological distress (Flett et al., 2002; O’Connor et al., 2007) and perfectionism to be positively related to obsessive-compulsive rumination symptoms (e.g., Rheaume et al., 2000). Correlations between PI subscales and the MPS have exhibited the intended conceptual similarities (Hill et al., 2004). The PI was used because it captures a wide range of perfectionism dimensions with one measurement instrument and thus redundancies between similar dimensions assessed with different tools can be avoided.

To investigate the factorial structure of the German PI, confirmatory factor analyses (CFA) were conducted with items as indicators. Model 1 consisted of a single first-order factor, Model 2 consisted of eight correlated first-order factors, and Model 3 consisted of two correlated second-order factors. According to Hill et al. (2004), Models 2 and 3 might be expected to fit the data reasonably well. Model 4 was evaluated as a two-factorial correlated bifactor model. It consisted of two general factors (perfectionistic strivings and perfectionistic concerns) and their respective specific factors. Model 5 was a bifactor model with one general factor and 8 specific factors. This bifactor model was of focal interest as a possible alternative representation of the PI’s latent structure and to gain clear information about how much observed indicator variance is explained by a common general factor or by distinct specific factors.

To further investigate the validity of the bifactor model, correlations with related constructs were assessed. Differential correlations for the general and the specific factors were expected to prove the bifactor model’s validity. Referring to the dual process model (Slade and Owens, 1998) and the literature on positive and negative outcomes associated with perfectionistic strivings and perfectionistic concerns, we expected positive correlations between perfectionistic concerns and fear of failure as well as neuroticism, and negative correlations with self-efficacy. We also expected positive correlations between perfectionistic strivings and hope for success, conscientiousness, and self-efficacy.

## Materials and Methods

### Sample and Procedure

A sample of *N* = 481 participants (316 women, 48.9% with a university or university of applied sciences degree) between the ages of 17 and 75 years (*M* = 35.70, *SD* = 12.66) completed an online questionnaire provided via the Unipark platform (EFS Survey Software, Globalpark). The link to the questionnaire was published on social community networks and via mail distribution lists.

### Measures

The 59 item PI (Hill et al., 2004) measures eight perfectionism dimensions, of which four are considered as perfectionistic strivings (high standards for others, organization, planfulness, striving for excellence), and four are considered as perfectionistic concerns (concern over mistakes, need for approval, perceived parental pressure, rumination). The 5-point rating scale ranges from 1 (*strongly disagree*) to 5 (*strongly agree*). CFA supported a model with two highly correlated factors of perfectionistic strivings and perfectionistic concerns (Hill et al., 2004). Correlations of PI subscales with MPS subscales and other criterion measures, such as fear of negative evaluation, psychiatric distress, obsessive-compulsive symptoms, and social desirability, provided some evidence for the PI’s validity. Internal consistency was found to be high (Hill et al., 2004), with Cronbach’s α ranging between 0.83 (high standards for others) and 0.91 (organization). For the purpose of comparison Cronbach’s α was calculated for the following subscales without checking the assumption of essential tau-equivalence first. For the PI’s subscales Cronbach’s α (see **Table 1**) ranged between 0.81 (striving for excellence) and 0.95 (perceived parental pressure) in the current sample.

**Table 1:** Correlations among Subscales, Internal Consistencies (Cronbach’s α), means, and standard deviations of the German PI, compared to Hill et al.’s (2004) PI results (in parentheses)

	Scale	HSO	O	P	SE	CM	NA	PP	R	PI
**Perfectionistic strivings**	High standards for others (HSO)	0.85 (0.83)								
	Organization (O)	0.20 (0.42)	0.87 (0.91)							
	Planfulness (P)	0.21 (0.29)	0.35 (0.49)	0.86 (0.86)						
	Striving for excellence (SE)	0.47 (0.49)	0.24 (0.59)	0.39 (0.39)	0.81 (0.85)					
**Perfectionistic concerns**	Concern over mistakes (CM)	0.31 (0.52)	0.05 (0.18)	0.30 (0.27)	0.55 (0.46)	0.90 (0.86)				
	Need for approval (NA)	0.22 (0.63)	0.04 (0.18)	0.34 (0.63)	0.49 (0.33)	0.80 (0.63)	0.88 (0.87)			
	Perceived parental pressure (PP)	0.16 (0.33)	-0.07 (0.19)	0.04 (0.17)	0.26 (0.31)	0.36 (0.33)	0.27 (0.19)	0.95 (0.88)		
	Rumination (R)	0.24 (0.47)	0.03 (0.38)	0.39 (0.43)	0.60 (0.52)	0.78 (0.73)	0.79 (0.67)	0.32 (0.34)	0.91 (0.87)	
	Total PI	0.52 (0.66)	0.33 (0.59)	0.56 (0.62)	0.76 (0.77)	0.82 (0.76)	0.78 (0.69)	0.52 (0.51)	0.82 (0.83)	0.94 (0.83)
	*M*	3.04 (2.83)	3.10 (3.50)	3.28 (3.40)	3.32 (3.10)	2.58 (2.46)	3.03 (3.22)	2.32 (3.17)	2.87 (2.83)	23.55 (24.51)
	*SD*	0.72 (0.78)	0.79 (0.86)	0.71 (0.76)	0.72 (0.80)	0.85 (0.75)	0.82 (0.75)	1.08 (0.89)	0.89 (0.82)	4.21 (4.40)

*Cronbach’s α entered on the main diagonal. All coefficients larger than 0.16 are significant, *p* < 0.01.*

In order to provide a German version of the PI, three psychology academics independently translated the original PI into German and agreed on an initial version, by discussing and selecting the most appropriate translation for each item. A professional native-speaking translator re-translated this version into English and compared it to the original version. On the advice of the native speaker, some minor corrections were made and the final version was used in the present study.

The short version of the Achievement Motives Scale (Lang and Fries, 2006) consists of two subscales, hope for success and fear of failure, each measured by five items. Internal consistency of the subscales was found to be high, with Cronbach’s α ranging between 0.71 and 0.88 (Lang and Fries, 2006). In the current sample α was 0.91 for both subscales.

To assess neuroticism and conscientiousness, the Big Five Inventory – short version (BFI-K; Rammstedt and John, 2005) was applied. The BFI-K is a German adaptation of the Big Five Inventory (John et al., 1991, 2008), with Cronbach’s α of the subscales ranging between 0.64 and 0.92 (Rammstedt and John, 2005). In the present study, we used the subscales neuroticism (α = 0.85) and conscientiousness (α = 0.67), each consisting of four items.

The General Self-Efficacy Scale (Schwarzer and Jerusalem, 1981–2014, Schwarzer and Jerusalem, 1995) was applied to assess the perceived competence expectation in handling challenging situations. The scale consists of ten items, with Cronbach’s α of the German version ranging between 0.80 and 0.90 (Schwarzer and Jerusalem, 1981–2014). The scale is widely used in empirical research and available in several languages. In the current sample α was 0.92.

To ease questionnaire handling, the rating scales of all scales were aligned with the PI to a 5-point rating scale.

### Data Analysis

We investigated the bivariate correlations between the PI subscales and the criterion measures with SPSS version 22. Using M*plus* version 7.2 (Muthén and Muthén, 1998–2012), the factorial structure of the PI was analyzed by means of CFA. As data deviated from normality, the robust maximum likelihood estimator was used in order to obtain robust standard error estimates (Rhemtulla et al., 2012). Model fit was evaluated by χ^2^-value, χ^2^/*df*, root mean square error of approximation (RMSEA), comparative fit index (CFI), Tucker-Lewis index (TLI), standardized root mean square residual (SRMR), and Akaike information criterion (AIC) for model comparison. Common cut-off values were used. Descriptive fit measures indicated good fit if χ^2^/*df* ≤ 2, RMSEA ≤ 0.05, CFI ≥ 0.97, TLI ≥ 0.97, and SRMR ≤ 0.05. Acceptable fit was indicated if χ^2^/*df* ≤ 3, RMSEA ≤ 0.08, CFI ≥ 0.95, TLI ≥ 0.95, and SRMR ≤ 0.10 (Schermelleh-Engel et al., 2003).

Schematic representations of the alternative models considered in this study are presented in **Figure 1**. Model 1, with a single first-order factor, was evaluated with all items loading on a single perfectionism factor. In Model 2, with eight correlated first-order factors, the items of each PI subscale loaded on one of the eight subscale factors. In the higher-order model (Model 3), items of each subscale loaded on their respective first-order factors. The eight first-order factors were expected to measure two correlated second-order factors, with first-order factors representing either perfectionistic strivings or perfectionistic concerns. According to Hill et al. (2004), the subscales high standards for others, organization, planfulness, and striving for excellence were assigned to perfectionistic strivings, and the subscales concern over mistakes, need for approval, perceived parental pressure, and rumination were assigned to perfectionistic concerns. The two-factorial bifactor model (Model 4) consisted of two correlated general factors and eight specific factors. Items measuring perfectionistic strivings loaded on a general perfectionistic strivings factor and additionally on one of four specific strivings factors. Items measuring perfectionistic concerns loaded on a general perfectionistic concerns factor and additionally on one of four specific concerns factors. The specific factors were uncorrelated. In the bifactor model (Model 5), with a general and eight specific factors, all items loaded on the general factor, and each item additionally loaded on one of eight specific subscale factors. As a canonical bifactor model, the general and the specific factors were assumed to be orthogonal.

For the identification of first-order models (Models 1, 2, 4, and 5), the latent factor variances were fixed to 1.0. For the second-order model (Model 3), the latent second-order factor variance and one factor loading on each first-order factor were fixed to 1.0.

We calculated coefficient omega (ω), coefficient omega subscale (ω_S_), coefficient omega hierarchical (ω_H_), and coefficient omega hierarchical subscale (ω_HS_) to judge the amount of explained variance due to the general factor and the specific factors (see Reise et al., 2013, for an overview of these coefficients).

Coefficient omega (ω; McDonald, 1999) is a reliability estimate based on the factor loadings of a CFA model. Omega estimates the proportion of variance in the observed scores explained by all sources of common variance included in the factor model. In a bifactor model, sources of common variance are the general factor and the specific factors (Reise et al., 2013).

Within the bifactor framework, coefficient omega hierarchical (ω_H_) estimates the proportion of variance in the observed scores that can be attributed to the general factor, while treating variance explained by the specific factors as unexplained measurement error (McDonald, 1999; Reise et al., 2013; Rodriguez et al., 2016). The ratio ω_H_/ω gives the amount of explained variance in the observed scores that can be attributed to the general factor, while the difference ω – ω_H_ gives the amount of explained variance in the observed scores that can be attributed to the specific factors (Rodriguez et al., 2016). Coefficient omega subscale (ω_S_) estimates the proportion of variance in the observed scores explained by the general and the specific factor. Coefficient omega hierarchical subscale (ω_HS_) estimates the reliability of a subscale score after controlling for the variance explained by the general factor (Reise et al., 2013), that is, high values indicate a large amount of unique variance associated with a specific factor.

Confidence intervals for hierarchical omega coefficients were calculated according to Raykov and Marcoulides (2011, p. 167).

### Preliminary Analysis

In a preliminary analysis, the comparability of our German translation of the PI with the original version was examined. Correlations among PI subscales, internal consistencies, means, and standard deviations are listed in **Table 1**. For the purpose of comparison across studies, we used Cronbach’s α as a measure of reliability for the manifest subscale scores, although other measures such as coefficient ω (McDonald, 1999) are often more informative because of the strict assumptions associated with α. Cronbach’s α indicated high internal consistencies of the subscales, ranging between 0.81 and 0.95. Means and standard deviations were comparable to the English version, and the overall correlation pattern was quite similar.

In contrast to Hill et al.’s (2004) findings, organization did not correlate with the perfectionistic concerns subscales. Striving for excellence, however, was substantially related to the perfectionistic concerns subscales and exhibited stronger correlations with concern over mistakes, need for approval, and rumination, than with perfectionistic strivings subscales. Correlations between perfectionistic strivings subscales were slightly lower, while correlations between perfectionistic concerns subscales were slightly higher for the German PI, compared to Hill et al.’s (2004) results. The second-order factors in Model 3 exhibited a strong positive correlation (*r* = 0.67), comparable to the correlation reported by Hill et al. (2004, *r* = 0.69). Taken as a whole, the overall pattern of descriptive statistics was comparable to the original version.

## Results

### A Bifactor Model of the German PI

The results of the CFA showed that the discrepancy between the model-implied covariance matrix and the empirical covariance matrix was significant for all models, as indicated by the χ^2^ statistic (see **Table 2**). We further investigated descriptive fit measures in order to evaluate the relative fit of the models. As expected, severe model misfit was found for Model 1, with a single first-order factor. Model 2, with eight correlated first-order factors, as well as Model 3, with two correlated second-order factors (*r* = 0.67), showed acceptable fit, with a χ^2^/*df*-ratio of 2.57 and 2.60, RMSEA = 0.06, and SRMR ≥ 0.08, respectively, while CFI and TLI indicated misfit.

**Table 2:** Summary of fit statistics for Alternative Confirmatory Factor models

Model	χ^2^	*df*	χ^2^/*df*	RMSEA	CFI	TLI	SRMR	AIC
(1) Single factor, 1st-order	11116.64^∗∗^	1652	6.73	0.11	0.43	0.41	0.14	77039.36
(2) 8 correlated factors, 1st-order	4165.86^∗∗^	1624	2.57	0.06	0.85	0.84	0.08	69490.52
(3) 2 correlated factors, 2nd-order	4279.27^∗∗^	1643	2.60	0.06	0.84	0.84	0.09	69580.19
(4) 2 correlated bifactors model	3642.07^∗∗^	1592	2.29	0.05	0.88	0.87	0.07	68975.51
(4a) 2 correlated bifactors-(*S* - 1) model	3955.55^∗∗^	1606	2.46	0.06	0.86	0.85	0.08	69292.24
(5) Bifactor model	3655.19^∗∗^	1593	2.29	0.05	0.88	0.87	0.08	68995.22
(5a) Bifactor-(*S* – 1) model	3782.08^∗∗^	1601	2.36	0.05	0.87	0.86	0.08	69123.59

*^∗∗^*p* < 0.01.*

When bifactor models (Models 4 and 5, see **Table 2**) were analyzed, several factor loadings on specific perfectionistic concerns factors were low or non-significant (e.g., items CM1, CM2, CM3, CM5, NA1, NA2, and R1 in Model 5). This pattern indicated the problem of collapsing factors that commonly occur in empirical applications of bifactor models (or multitrait-multimethod models in general; Geiser et al., 2015; Eid et al., 2016). As Geiser et al. (2015, p. 14) explicate, “if one or more specific factors collapse, the general factor becomes specific to the set of indicators for which the specific factor(s) collapsed.” This problem is likely to occur when specific factors are treated as interchangeable facets rather than structurally different facets (Geiser et al., 2015; Eid et al., 2016). With collapsing items the meaning of the general factor changes. When considering, for example, Model 5 with one general factor and eight specific factors, the general factor cannot be interpreted as a general perfectionism factor. Instead, the general factor becomes specific to the set of indicators for which the specific factor collapsed. Therefore, the general factor of Model 5 comprises primarily the true-score variance of the items measuring concern over mistakes. In this case, applying the bifactor-(*S* – 1) model is recommended in order to give the general factor a clear meaning. This model is called bifactor-(*S* – 1) model because there is one specific factor (*S*) less than facets considered (Geiser et al., 2015; Eid et al., 2016). Through this modification the discarded specific factor has the function of a reference facet for the general factor and the general factor becomes well-defined concerning its content.

Following this recommendation, bifactor-(*S* – 1) models were analyzed (Models 4a and 5a). In Model 4a, items measuring concern over mistakes were restricted to load onto the general perfectionistic concerns factor only and items measuring striving for excellence were restricted to load onto the general perfectionistic strivings factor only. Thus, the general factors were defined by their reference facets. This model showed acceptable fit, with a χ^2^/*df*-ratio of 2.46, RMSEA = 0.06, and SRMR = 0.08. However, the latent correlation between the general perfectionistic concerns and the general strivings factor was high (*r* = 0.72), indicating high construct overlap and a substantial amount of shared variance.

In the bifactor-(*S* – 1) model with only one general factor (Model 5a) all items measuring concern over mistakes were restricted to load onto the general factor only. In this model, the general factor was now explicitly modeled with concern over mistakes as the reference facet. Compared to Model 4a with two correlated bifactors, model fit slightly improved (χ^2^/*df*-ratio of 2.36, RMSEA = 0.05, and SRMR = 0.08). This model showed the highest CFI and TLI values, and the lowest AIC, when Models 4 and 5 were not considered because of the problem of collapsing factors.

Thus, the bifactor-(*S* – 1) model with concern over mistakes as the reference facet for the general factor was preferred. For all other facets a specific factor was defined as a residual factor which represents that part of a facet that is not shared with the general factor.

**Table 3** displays the factor loadings for this bifactor-(*S* – 1) model. All items measuring perfectionistic strivings showed significant loadings on their respective specific factors. That means that these specific factors explained a substantial amount of indicator variance. Loadings of these perfectionistic strivings items (except items SE4 and SE5) on the general factor were either considerably lower than those on the specific factors or even non-significant. For example, four of the organization items and one planfulness item had non-significant loadings on the general factor, and two items (HSO1 and O1) showed significant (but small) negative loadings on the general factor.

**Table 3:** Standardized Factor Loadings for the Bifactor-(*S* – 1) Model with One General Factor using Concern over Mistakes as the Reference Facet, and (8 – 1) Specific Factors.

	Item	General	HSO	O	P	SE	CM	NA	PP	*R*
**Perfectionistic strivings**	HSO1	-0.12	0.51							
	HSO2	0.36	0.66							
	HSO3	0.11	0.61							
	HSO4	0.26	0.61							
	HSO5	0.25	0.70							
	HSO6	0.31	0.67							
	HSO7	0.38	0.61							
	O1	-0.18		0.58						
	O2	0.12		0.62						
	O3	-0.04 n.s.		0.85						
	O4	0.18		0.75						
	O5	0.16		0.60						
	O6	0.07 n.s.		0.74						
	O7	0.03 n.s.		0.67						
	O8	-0.01 n.s.		0.67						
	P1	0.05 n.s.			0.72					
	P2	0.20			0.61					
	P3	0.21			0.82					
	P4	0.42			0.55					
	P5	0.40			0.48					
	P6	0.23			0.75					
	P7	0.31			0.49					
	SE1	0.25				0.62				
	SE2	0.29				0.66				
	SE3	0.30				0.50				
	SE4	0.54				0.40				
	SE5	0.68				0.29				
	SE6	0.48				0.55				
**Perfectionistic concerns**	CM1	0.72					0			
	CM2	0.71					0			
	CM3	0.74					0			
	CM4	0.64					0			
	CM5	0.74					0			
	CM6	0.80					0			
	CM7	0.61					0			
	CM8	0.77					0			
	NA1	0.60						-0.03 n.s.		
	NA2	0.62						0.07 n.s.		
	NA3	0.42						0.21		
	NA4	0.62						0.48		
	NA5	0.59						0.18		
	NA6	0.71						0.55		
	NA7	0.74						0.23		
	NA8	0.73						0.54		
	PP1	0.32							0.82	
	PP2	0.28							0.85	
	PP3	0.34							0.78	
	PP4	0.23							0.87	
	PP5	0.42							0.66	
	PP6	0.36							0.84	
	PP7	0.35							0.79	
	PP8	0.30							0.66	
	R1	0.68								0.09 n.s.
	R2	0.67								0.27
	R3	0.74								0.18
	R4	0.70								0.53
	R5	0.75								0.50
	R6	0.73								0.39
	R7	0.61								0.29
	ω or ω_S_	0.96	0.85	0.88	0.87	0.83	0.90	0.89	0.96	0.91
	ω_H_ or ω_HS_	0.80	0.76	0.88	0.74	0.49	—	0.15	0.82	0.16
	95% CI-LB	0.78	0.69	0.86	0.67	0.39	—	0.11	0.74	0.12
	95% CI-UB	0.83	0.81	0.90	0.79	0.55	—	0.20	0.87	0.22

*HSO, high standards for others; O, organization; P, planfulness; SE, striving for excellence; CM, concern over mistakes; NA, need for approval; PP, perceived parental pressure; R, rumination; ω, coefficient omega; ω_*S*_, coefficient omega subscale; ω_**H**_, coefficient omega hierarchical; ω_*HS*_, coefficient omega hierarchical subscale; 95% CI-LB, 95% confidence interval for ω_*H*_ or ω_*HS*_, lower boundary; 95% CI-UB; 95% confidence interval for ω_*H*_ or ω_*HS*_, upper boundary.*

The items measuring perfectionistic concerns showed a different factor loading pattern. All items had higher loadings on the general factor than on their specific factors, with the exception of items measuring perceived parental pressure, which loaded higher on their specific factor than on the general factor.

Factor loadings only differed marginally compared to the results obtained from the original bifactor solution with eight specific factors (not reported in detail here).

We calculated coefficient omega (ω), coefficient omega subscale (ω_S_), coefficient omega hierarchical (ω_H_), and coefficient omega hierarchical subscale (ω_HS_; see **Table 3**). Coefficient ω was high (0.96, 95% CI [0.96,0.97]), which means that 96% of the total variance was explained by the general factor and the specific factors.

The amount of explained variance by the general and the specific factor for each subscale was high with ω_S_ ranging between 0.83 (striving for excellence) and 0.96 (perceived parental pressure).

In order to disentangle the different sources of variance, the hierarchical omega coefficients provide the relevant information.

The proportion of variance in the observed scores that can be attributed to the general factor, while treating variance explained by the specific factors as unexplained measurement error, was high (ω_H_ = 0.80, 95% CI [0.78,0.83]).

The ratio ω_H_/ω was 0.80/0.96 = 0.83 and indicated that the explained variance in the observed scores can be attributed to the general factor to a large degree. The difference ω – ω_H_ was 0.96 -0.80 = 0.16 (95% CI [0.14,0.19]), indicating that only a small amount of explained variance in the observed scores could be attributed to the specific factors.

The specific strivings factors high standards for others, organization, and planfulness had high values for coefficient ω_HS_ ranging between 0.74 and 0.88. This means that a large amount of unique variance was associated with these specific factors. Only for the specific factor striving for excellence, ω_S_ was moderate (0.49). These perfectionistic strivings factors were controlled for the impact of the general factor, which was defined by the reference facet concern over mistakes. Thus, the specific factors can be considered as pure factors of specific perfectionistic strivings, uncontaminated by perfectionistic concerns.

For the specific concerns factors measuring need for approval (ω_HS_ = 0.15) and rumination (ω_HS_ = 0.16) only a small amount of the items’ unique variance was associated with these factors. Coefficient ω_HS_ was high for the specific factor perceived parental pressure (0.82). Thus, a considerable amount of explained variance in observed scores was due to this specific factor.

### Correlations With Criterion Measures

We first used manifest subscale scores of the PI, as usually applied in empirical research, in correlation analysis with manifest scale scores of criterion measures (see **Table 4**). As expected, the subscales measuring perfectionistic concerns were positively correlated with fear of failure and neuroticism (*r* ranging between 0.20 and 0.70), and they were negatively correlated with self-efficacy (*r* ranging between -0.17 and -0.41).

**Table 4:** Correlations of German PI Manifest Subscales and Total Scores with Criterion Measures.

	Subscale	Hope for success	Fear of failure	Neuroticism	Conscientiousness	Self-efficacy
**Perfectionistic strivings**	HSO_subscale_	0.26ˆ**	0.05	0.08	0.22^∗∗^	0.12^∗∗^
	O_subscale_	0.03	0.01	-0.02	0.45^∗∗^	0.09^∗^
	P_subscale_	0.00	0.22ˆ**	0.24^∗∗^	0.18^∗∗^	-0.09^∗^
	SE_subscale_	0.25ˆ**	0.26ˆ**	0.33^∗∗^	0.31^∗∗^	-0.05
**Perfectionistic concerns**	CM_subscale_	-0.04	0.64ˆ**	0.62^∗∗^	-0.10^∗∗^	-0.40^∗∗^
	NA_subscale_	0.00	0.67ˆ**	0.70^∗∗^	-0.10^∗∗^	-0.41^∗∗^
	PP_subscale_	-0.01	0.20ˆ**	0.24^∗∗^	-0.03	-0.17^∗∗^
	R_subscale_	-0.03	0.62ˆ**	0.70^∗∗^	-0.08	-0.39^∗∗^
	Perfectionism_total_	0.08	0.54ˆ**	0.58^∗∗^	0.14^∗∗^	-0.27^∗∗^

*HSO, high standards for others; O, organization; P, planfulness; SE, striving for excellence; CM, concern over mistakes; NA, need for approval; PP, perceived parental pressure; R, rumination. ^∗^*p* < 0.05, ^∗∗^*p* < 0.01.*

For the perfectionistic strivings subscales, results were ambiguous. Positive associations with conscientiousness were confirmed (*r* ranging between 0.18 and 0.45), and the subscales high standards for others and striving for excellence correlated with hope for success (*r* = 0.26 and 0.25, respectively). However, we did not find substantial positive correlations between perfectionistic strivings subscales and self-efficacy, with the exception of high standards for others (*r* = 0.12). Contrary to what would have been expected, the subscales planfulness and striving for excellence correlated positively with fear of failure and neuroticism (*r* ranging between 0.22 and 0.33).

In order to investigate the validity of the bifactor-(*S* – 1) model, the latent general and specific factor scores obtained from this model were also correlated with manifest scale scores of criterion measures (see **Table 5**). As expected, we now found positive correlations between the specific factors of perfectionistic strivings and conscientiousness (*r* ranging between 0.26 and 0.48) and self-efficacy (*r* ranging between 0.10 and 0.26). High standards for others and striving for excellence correlated positively with hope for success (*r* = 0.28 and 0.35, respectively), and negatively with fear of failure (*r* = -0.17) and neuroticism (*r* = -0.15 and -0.12, respectively).

**Table 5:** Correlations of Specific and General Factor Scores from the Bifactor-(*S* – 1) Model with Criterion Measures.

	Factor	Hope for success	Fear of failure	Neuroticism	Conscientiousness	Self-Efficacy
**Perfectionistic strivings**	HSO_specific_	0.28ˆ**	-0.17ˆ**	-0.15ˆ**	0.26ˆ**	0.26ˆ**
	O_specific_	0.01	-0.02	-0.05	0.46ˆ**	0.11ˆ*
	P_specific_	0.03	-0.05	-0.03	0.27ˆ**	0.10ˆ*
	SE_specific_	0.35ˆ**	-0.17ˆ**	-0.12ˆ*	0.48ˆ**	0.25ˆ**
**Perfectionistic concerns**	NA_specific_	0.06	0.14ˆ**	0.18ˆ**	0.01	-0.03
	PP_specific_	0.00	-0.06	-0.02	0.01	-0.02
	R_specific_	-0.04	0.14ˆ*	0.24ˆ**	-0.04	-0.07
	Perfectionism_general_	-0.02	0.68ˆ**	0.71ˆ**	-0.10ˆ*	-0.43ˆ**

*HSO, high standards for others; O, organization; P, planfulness; SE, striving for excellence; NA, need for approval; PP, perceived parental pressure; R, rumination. ^∗^*p* < 0.05, ^∗∗^*p* < 0.01.*

Most of the original information of need for approval and rumination was captured by the general factor with concern over mistakes as the reference facet. The specific factors need for approval and rumination were residual factors capturing specific variance when the overlap with the general factor was controlled for. Thus, these specific factors did not explain the criterion measures over and above the general factor, except for small but significant correlations between the specific factors need for approval and rumination and fear of failure and neuroticism (*r* ranging between 0.14 and 0.24).

This lack of substantial relationships was due to the fact that a large amount of information of the items measuring perfectionistic concerns was captured by the general factor which showed the correlation pattern expected for perfectionistic concerns. The general perfectionism factor exhibited significant positive relations with fear of failure (*r* = 0.68) and neuroticism (*r* = 0.71), as well as a negative correlation with self-efficacy (*r* = -0.43).

Compared to the application of latent factor scores, the manifest scale scores showed smaller correlations between perfectionistic strivings and positive criterion measures (hope for success, conscientiousness, and self-efficacy), and we found unexpected positive correlations between perfectionistic strivings subscales and negative criterion measures.

To sum up, the pattern of correlations for perfectionistic strivings was more comprehensible when latent factor scores of the bifactor-(*S* – 1) model were used instead of manifest subscale scores, and the general perfectionism factor correlated with the criterion variables in a manner as expected for perfectionistic concerns.

## Discussion

The evaluation of a bifactor model of perfectionism in order to disentangle the common variance of perfectionistic strivings and perfectionistic concerns was of focal interest for the present study. We investigated whether a bifactor model of perfectionism might be a reasonable and viable alternative to existing factor models. As our results showed, the bifactor model with a single general factor and S (*S* = 8) specific factors as well as the bifactor model with two correlated general factors and 8 specific factors both encountered the problem of collapsing specific factors. This problem is well-known from multitrait-multimethod models and similarly structured methodology which result in solutions where at least one method or specific factor shows non-significant factor loadings (cf. Eid et al., 2008; Geiser et al., 2015; Eid et al., 2016). Collapsing factors can cause problems because the meaning of the general factor changes. In this case the general factor cannot be interpreted as a factor comprising the common variance of all items, but it becomes specific to the items of the collapsed factor. Therefore, specifying a bifactor-(*S* – 1) model by taking one facet as the reference facet is recommended, as was done in Model 5a. When the general factor is defined as the common factor of the reference facet, i.e., concern over mistakes, the meaning of the general factor does not change when facets are added or removed.

Confirmatory factor analysis resulted in an acceptable fit for the bifactor-(*S* – 1) model and correlation analyses provided initial evidence for the bifactor model’s validity. Our results demonstrated some advantages of the bifactor model compared to first-order and second-order factor models. According to the *S* – 1 approach, concern over mistakes was chosen as a reference facet and thus the general factor in this model represented concern over mistakes. The general factor explained a huge amount of indicator variance, especially for items intended to measure perfectionistic concerns. The correlation between perfectionistic strivings and perfectionistic concerns that has been reported in previous studies (e.g., Hill et al., 2004; Dunkley et al., 2012) can be explained by the general factor. This general factor influences almost all items of the PI and thus, can be interpreted as their common cause. Besides this general factor, perfectionistic strivings factors remained as specific factors, reliably explaining substantial indicator variance. However, the general factor explained a substantial amount of variance of items measuring striving for excellence, i.e., the core facet of perfectionistic strivings. Therefore, the bifactor-(*S* – 1) model was regarded as useful to disentangle the common variance of perfectionistic strivings and perfectionistic concerns at the item level. The general factor’s influence was lowest for high standards for others and organization. With regard to the perfectionistic concerns factors, only perceived parental pressure remained as a specific factor while the other three factors did not differ from the general factor substantially.

In contrast to previous research (Hill et al., 2004; Cruce et al., 2012; Smith and Saklofske, 2016), we used items as indicators of first-order factors instead of composite scores to assess the factorial structure of the German PI. According to the findings of Hill et al. (2004) and Cruce et al. (2012), a model with eight correlated first-order factors representing the subscales of the PI, and a model with two second-order factors of perfectionistic strivings and perfectionistic concerns would have been expected to fit the data well. However, these models exhibited only moderate fit for the German PI. We found a strong positive correlation between the two higher-order factors of perfectionistic strivings and perfectionistic concerns (*r* = 0.67), as well as between two correlated bifactors (*r* = 0.72) which were similar to the correlation (*r* = 0.69) reported by Hill et al. (2004).

We found support for the bifactor-(*S* – 1) model of the PI with concern over mistakes as the reference facet. This model fitted the data better than the first-order and second-order factor models and better than a model with two correlated bifactors. This could be expected since the bifactor model is a less restrictive model. Although some misfit was found for the bifactor-(*S* – 1) model as well, the AIC was lowest for this model and the RMSEA and χ^2^/df-ratio were satisfying. It is challenging to achieve results that meet with usual cut-off criteria for model fit indices for a large model based on items as indicators. In this study, model estimation was based on the huge number of 59 items. Some error covariances could be expected as well, for example due to similar wording. However, it was not intended to improve model fit by allowing error covariances because these are irrelevant for the actual model structure.

The factor loading pattern of the bifactor-(*S* – 1) model showed that the general factor accounted for nearly all of the common variance in the perfectionistic concerns items with the exception of perceived parental pressure. The specific factors need for approval and rumination contained too little true score variance to be viewed as specific measures of these constructs after controlling for the general factor. Only for perceived parental pressure 82% of reliable variance was independent of the general factor.

The specific perfectionistic strivings factors high standards for others, organization, and planfulness, controlled for the impact of the general factor, each explained a substantial amount of reliable variance (ω_HS_ > 0.74), and the specific factor striving for excellence still accounted for 49% of the reliable variance of this scale.

The items measuring organization were unrelated with the general factor. This result is consistent with previous findings for the MPS-F. The organization subscale was found to correlate only weakly with the other perfectionism subscales (Altstötter-Gleich and Bergemann, 2006). Organization was even excluded from the total perfectionism score (Frost et al., 1990) and found to be a separate factor besides perfectionistic strivings and concerns (Kim et al., 2015). However, the organization subscale of the PI may be used to measure one of the multidimensional aspects of perfectionistic strivings.

Our results demonstrated that it is possible to differentiate between perfectionistic strivings and perfectionistic concerns by means of a bifactor-(*S* – 1) model. We found some initial evidence supporting the validity of the suggested bifactor solution in correlation analysis with the criterion measures of achievement motivation, neuroticism, conscientiousness, and self-efficacy. The general factor correlated with the criteria in a way one would have expected for perfectionistic concerns, while the specific perfectionistic concerns factors had little predictive value over and above the general factor with regard to the selected criteria. Since the specific factors controlled for the common variance due to the general factor, the specific perfectionistic strivings factors correlated with criterion measures in a comprehensible manner and met our expectations better compared to correlations based on manifest subscale scores. When subscale scores were used, the confounding of perfectionistic strivings and perfectionistic concerns in the subscales intended to measure perfectionistic strivings became apparent in ambiguous correlations with criterion measures.

The PI provides a tool for measuring a broad range of perfectionism dimensions. However, our findings indicated that in empirical research, manifest subscale scores should be interpreted with caution, due to the possible contamination of perfectionistic strivings with perfectionistic concerns. Instead of manifest subscale scores, latent factor scores of a bifactor-(*S* – 1) model might be more appropriate for evaluating relations with criterion measures and might have more predictive value. To summarize, the results of this study yielded consistent evidence in favor of the bifactor-(*S* – 1) model of the German PI compared with the first-order and second-order oblique models investigated so far.

Smith and Saklofske (2016) also favored a bifactor model of perfectionism in their study. They suggested a bifactor model with one general factor and two specific factors of perfectionistic strivings and perfectionistic concerns, using subscales of different perfectionism measures as indicators. In contrast to our bifactor-(*S* – 1) model, they did not use a reference facet for the general factor and did not explicitly define the general factor’s conceptual meaning. In our bifactor-(*S* – 1) model, however, the general factor represented concern over mistakes.

In contrast to using partial correlations, the bifactor-(*S* – 1) model provides some advantages that can address the problem of shared variance between perfectionistic strivings and perfectionistic concerns. First, the perfectionistic strivings factors are residual factors and therefore do not share common variance with the general factor of the bifactor-(*S* – 1) model (thus, they do not share common variance with concern over mistakes as the reference facet of the general factor). Instead of controlling for deficiencies in item construction at the level of scale composite scores, shared variance of perfectionistic strivings and concerns can be partialled out at the item level. Second, the bifactor-(*S* – 1) model offers the usual advantages associated with latent variable modeling in general, such as control of measurement error. Third, the bifactor-(*S* – 1) model clarifies which items share common variance captured by the general factor and which items remain indicators of a specific factor. This information might be used in order to refine the measurement instrument, but it also gives insight into the nature of the perfectionism factors as measured on a given scale.

### Limitations and Future Directions

However, some open questions concerning the suggested bifactor-(*S* – 1) model remain. First, as the subscales concern over mistakes, need for approval, and rumination were highly correlated (see **Table 1**), and as only few items supposed to measure these constructs captured unique variance after controlling for the variance explained by the general factor, the discriminant validity of these factors is questionable. Their content seems to be redundant to a large degree, indicating that the questionnaire may be shortened without losing valuable information. If further studies confirm the low discriminant validity of these factors, the dimensions of perfectionistic concerns may have to be reconsidered. Compared to the FMPS, including rumination as a further dimension of perfectionistic concerns does not seem to add any value. In empirical research, only one subscale is often used to measure perfectionistic concerns (e.g., concern over mistakes of the MPS-F; or socially prescribed perfectionism of the MPS-HF) and one subscale is often used to measure perfectionistic strivings (e.g., personal standards of the MPS-F; or self-oriented perfectionism of the MPS-HF). Our findings support this practice, if aspects of orderliness, planning and interpersonal perfectionistic expectations are irrelevant for the respective research question. Within the bifactor-(*S* – 1) model, perfectionistic concerns are represented in the general factor and the specific factor striving for excellence reflects perfectionistic strivings controlled for the impact of the general factor. However, a cross-validation of our findings is needed.

Second, the results of the bifactor-(*S* – 1) model raise questions concerning the construct and measurement of perfectionism in general. As shown previously when partial correlation analyses were conducted on the subscales of the MPS, the intended perfectionistic strivings dimensions contained some content of perfectionistic concerns (Stoeber and Otto, 2006) comparable to our own findings. Stoeber and Gaudreau (2017) provide a useful non-technical explanation of partialled effects for perfectionistic strivings and perfectionistic concerns. However, due to the confounding of perfectionistic strivings and perfectionistic concerns at the item level, the measurement of pure perfectionistic strivings might be questioned. Thus, the grouping of subscales or factors into perfectionistic strivings and perfectionistic concerns may oversimplify the structure of the underlying construct. In order to gain a clearer understanding of the underlying structure, bifactor-(*S* – 1) modeling should be considered for other widely used measures (e.g., MPS-F, MPS-HF) as well. Moreover, future research is needed to investigate whether the findings of the present study can be generalized to other populations, especially to clinical populations.

As outlined above, some questions concerning the bifactor-(*S* – 1) model remain open. However, the proposed model accentuates the need for further investigation of the measurement and structure of perfectionism, in order to gain a clearer understanding of the perfectionism construct and the underlying factorial structure.

## Ethics Statement

The Deutsche Gesellschaft für Psychologie (German Psychological Association) provides ethical standards as guidelines for psychological practice and research. The study was conducted in accordance with these guidelines.

## Author Contributions

JG was mainly in charge of the conceptual design of the study, data collection, data analysis, and drafted the manuscript. JG and KS-E contributed to translation of the Perfectionism Inventory and to data collection. JG, KS-E, and AK contributed to interpretation of the data, critical revision of the manuscript, and all authors approved the final version.

## Conflict of Interest Statement

The authors declare that the research was conducted in the absence of any commercial or financial relationships that could be construed as a potential conflict of interest.
